# Physical activity, a modulator of aging through effects on telomere biology

**DOI:** 10.18632/aging.103504

**Published:** 2020-06-23

**Authors:** Maria Donatella Semeraro, Cassandra Smith, Melanie Kaiser, Itamar Levinger, Gustavo Duque, Hans-Juergen Gruber, Markus Herrmann

**Affiliations:** 1 Clinical Institute of Medical and Chemical Laboratory Diagnostics, Medical University of Graz, Graz, Austria; 2 Institute for Health and Sport (IHES), Victoria University, Melbourne, VIC, Australia; 3 Australian Institute for Musculoskeletal Science (AIMSS), University of Melbourne and Western Health, St Albans, VIC, Australia; 4 Department of Medicine-Western Health, Melbourne Medical School, The University of Melbourne, Melbourne, VIC, Australia

**Keywords:** telomeres, telomerase, physical activity, exercise, aging

## Abstract

Aging is a complex process that is not well understood but involves finite changes at the genetic and epigenetic level. Physical activity is a well-documented modulator of the physiological process of aging. It has been suggested that the beneficial health effects of regular exercise are at least partly mediated through its effects on telomeres and associated regulatory pathways. Telomeres, the region of repetitive nucleotide sequences functioning as a “cap” at the chromosomal ends, play an important role to protect genomic DNA from degradation. Telomeres of dividing cells progressively shorten with age. Leucocyte telomere length (TL) has been associated with age-related diseases. Epidemiologic evidence indicates a strong relationship between physical activity and TL. In addition, TL has also been shown to predict all-cause and cardiovascular mortality. Experimental studies support a functional link between aerobic exercise and telomere preservation through activation of telomerase, an enzyme that adds nucleotides to the telomeric ends. However, unresolved questions regarding exercise modalities, pathomechanistic aspects and analytical issues limit the interpretability of available data. This review provides an overview about the current knowledge in the area of telomere biology, aging and physical activity. Finally, the capabilities and limitations of available analytical methods are addressed.

## Introduction

Over the last 200 years average life expectancy in developed countries has more than doubled and is now above 80 years [1]. In numerous studies this linear increase is suggested to rise to an average life span of 100 years or more [2–4]. This dramatic increase in life expectancy was largely driven by changes in lifestyle, sanitation and a continuous improvement of health care [5]. As a result, the major causes of death have shifted from infectious disease to chronic age-related conditions [6, 7]. Today, cardiovascular disease (CVD), cancer and respiratory disease are the most common causes of death worldwide [8, 9]. Other lifestyle and age-related conditions such as musculoskeletal disease, diabetes and dementia are also increasing rapidly and thus impact the number of disability-adjusted life years (DALYs), calculated in a population as the sum of the Years of Life Lost (YLL) due to premature mortality and the Years Lost due to Disability (YLD) [10, 11]. Therefore, strategies to promote healthy aging have gained great interest in developed societies.

The process of aging remains incompletely understood. A better understanding of the complex and interrelated biological mechanisms of aging would help to develop interventions that delay the aging process. Environmental and lifestyle factors, such as physical activity, nutrition, stress and smoking, are major determinants of the aging process [12]. In particular, regular exercise is a safe and cost-effective way to reduce morbidity and premature mortality [13]. However, the molecular mechanisms that mediate the beneficial effects of exercise are incompletely understood and remain an area of active research. In numerous observational and intervention studies the preservation of telomeres, the protective end-caps of all chromosomes, has been proposed as an appealing putative mechanism that contributes partially to the beneficial health effects of physical activity [14]. This review aims to provide an overview on the current knowledge in the area of telomere biology, aging and physical activity. In addition, the capabilities and limitations of available analytical methods will be addressed.

## Structure and function of telomeres

The genetic information of eukaryotes is encoded in the deoxyribonucleic acid (DNA), which is packed in the chromosomes. With every division of mitotic cells a small fragment of DNA at the ends of every chromosome remains unreplicated due to a physiological phenomenon named the end-replication problem [15]. In order to prevent the loss of coding genetic information thousands of identical, non-coding oligonucleotides are attached to the ends of all chromosomes. These terminal non-coding DNA-regions are called telomeres. Human telomeres contain approximately 2,500 tandem copies of a simple hexanucleotide with the sequence 5'-TTAGGG_n_-3' [16]. For most of its length, telomeric DNA is double stranded. However, the last portion of 30–100 base pairs (bp) at the 3’-end of the G-rich strand is single-stranded. This G-rich overhang at the 3’-end is essential for telomere maintenance and capping [17, 18]. Telomeres give rise to a complex three-dimensional structure limiting the access of telomerase and DNA damage repair (DDR) enzymes to the free ends of each DNA-strand. This three-dimensional structure is achieved through the binding of a highly abundant protein complex, named shelterin, to the telomeric hexanucleotide sequence 5’-TTAGGG_n_-3‘. The shelterin complex is composed of the following six-subunits (see Table 1): telomeric repeat binding factor 1 (TRF1), telomeric repeat binding factor 2 (TRF2), TRF1-interacting nuclear protein 2 (TIN2), telomeric overhang binding protein 1 (POT1), TIN2 and POT1 interacting protein 1 (TPP1), and repressor-activator protein 1 (RAP1). TRF1 and TRF2 interact with the double-stranded telomeric DNA, whereas POT1 associates with single-stranded telomeric DNA [19]. Through interactions with the shelterin proteins the terminal telomere section flips backwards resulting in the formation of a looped structure (t-loop). Furthermore, the shelterin proteins aid in displacing a short section of double-stranded telomeric DNA so that the single stranded G-rich overhang at the 3´end can be interposed. This structure is referred to as “D-loop” and protects the free end of the DNA strand from recognition as a strand break, which would induce inappropriate repair processes.

**Table 1:** Shelterin complex, subunits and functions.

**Shelterin subunits**	**Function**	**References**
Telomeric repeat binding factor 1 (TRF1)- binds to the canonical TTAGGG double-stranded telomeric repeats	Determines the structure of telomeric ends, it is implicated in the generation of t-loops, and it controls the synthesis of telomeric DNA by telomerase	de Lange [20]
Telomeric repeat binding factor 2 (TRF2)- TRF1 paralog	Implicated in telomere protection and telomere length homeostasis	Takai et al. [21]; Artandi et al. [22]; Palm et al. [23]
TRF1-interacting nuclear protein 2 (TIN2)- can bridge TRF1 and TRF2/RAP1 complex and can also recruit the TPP1/POT1 heterodimer	Responsible for the recruitment of other shelterins, therefore implicated in telomere protection	Lei et al. [24]
Telomeric overhang binding protein 1 (POT1)- associates with the single-stranded TTAGGG repeats	The telomere length maintenance is exerted through the interaction between POT1 and the reverse-transcriptase ribonucleoprotein telomerase	Baumann et al. [25]; Loayza et al. [26]
TIN2 and POT1 interacting protein 1 (TPP1)	Required for the recruitment of telomerase to the DNA	van Steensel et al. [27]
Repressor-activator protein 1 (RAP1)- 1:1 complex with TRF2	In addition to its telomeric function, also implicated in the upregulation of energy metabolism as a modulator of the NF-κB signalling pathway	de Lange [20]; Teo et al. [28]

The interaction between the shelterin protein subunits is complex and has been investigated using mouse conditional knock-out cells for TRF1, TRF2, POT1, TPP1. It has been shown in several studies that shelterins prevent DNA damage response (DDR) activity at telomeres, chromosomal rearrangements and cell cycle arrest, thus demonstrating a role in maintaining telomere function and preserving genomic stability [17, 18, 29]. Through the binding of TRF1 and TRF2 to double-stranded telomeric TTAGGG_n_ repeats RAP1, TIN2, TPP1 and POT1 can be recruited. TIN2 can bridge TRF1 and TRF2/RAP1 complexes by binding to both proteins simultaneously. Furthermore, TIN2 associates with the TPP1/POT1 heterodimer, which is typically bound to single-stranded TTAGGG repeats [19, 24]. These intimate interactions result in the formation of a “capped” loop [20, 30, 31].

Telomere length (TL) varies greatly between species [32]. At birth, every human individual has a specific TL that ranges between 5 to 15 kb [33]. Throughout life telomeres shorten continuously with a rate between 20-50 bp due to the end-replication phenomenon, oxidative stress and other modulating factors [15, 33]. However, telomere shortening rates and consequently also average TL vary amongst different tissue types, which is at least partly explained by tissue-specific proliferation rates [34, 35]. In dividing cells, the end replication problem is an important driver of telomere shortening that can be modified by other factors, such as oxidative stress or inflammation [33]. In postmitotic cells instead, oxidative stress can directly damage telomeric DNA and drive cells into senescence [36, 37]. The TL of peripheral blood leucocytes (LTL) has gained substantial interest as a potential marker of biological age [17]. Mean LTL in adults is approximately 11 kb and declines with an annual rate of 30-35 bp. Telomere attrition is most pronounced during the first two years of life, which are characterized by rapid somatic growth [34, 35, 38, 39]. The shortening of telomeres is not a unidirectional process since the reverse-transcriptase telomerase is capable of adding new hexanucleotides to telomeric ends [40, 41]. However, most somatic cells do not express telomerase. Detectable levels of telomerase activity can typically be found in germ line and embryonic stem cells, immune cells and in cancer cells [42, 43]. Human telomerase is made up of two main components: telomerase reverse transcriptase (TERT) and telomerase RNA component (TERC) endowed with a complementary sequence of telomeric DNA (3’-AUCCC-5’), which serves as a template for telomere elongation [44]. It is important to note that telomerase expression does not necessarily parallel enzyme activity [44]. In the brain for example, TERT is expressed without detectable telomerase activity (TA) [45]. In contrast, PI3K/Akt and other factors can modulate TA independently from TERT expression [46]. In humans, the telomerase enzyme complex is completed by several associated proteins, including dyskeratosis congenita 1 (DKC1) and NOP10 ribonucleoprotein (NOP10), which are essential for the maintenance of telomere integrity [47, 48].

In order to add new TTAGGG hexanucleotides the enzyme needs access to the telomere ends, which are hidden in the complex three-dimensional telomere structure [31]. Therefore, telomeres can change their conformational status between an ‘open’ state, where the enzyme has substrate access, and a “closed” state that prevents telomerase action [49]. Shelterin proteins play a key role in regulating the conformational state of telomeres and thus modulate TA [27, 50]. The low number of TRF1 and POT1 binding sites on short telomeres drives the formation of an open state. Whereas longer telomeres, with more TRF1 and POT1 binding sites, typically assume a closed configuration [26]. In this way, telomerase can be efficiently directed to the shortest telomeres within a cell, and sufficiently long telomeres will not undergo any inappropriate lengthening [51, 52].

The importance of TL and TA in the aging process has been described by Rudolph et al. demonstrating that age-dependent telomere shortening, and genetic instability are associated with shortened life span and a reduced regenerative potential [53]. Several genetic disorders with mutations in loci encoding for shelterin and telomerase subunits have also been described, all of which have been characterized by an accelerated rate of telomere attrition [54–56]. Higher rates of leucocytes telomere attrition are also associated with elevated risk of coronary artery disease, myocardial infarction, heart failure and stroke [57]. Additionally, changes in LTL, shelterin expression and function have been linked to structural changes in the thoracic aorta vessel wall and the myocardium [58, 59]. Furthermore, shorter LTL is related to the increased severity of CVD [60–62]. Many factors contribute to the shortening of telomeres including the genetic background [56, 63–65], gender [66], socioeconomic status and consequent stress perceived [58, 67, 68], dietary behaviour (i.e. antioxidant intake, alcohol consumption etc.) [69–73], body mass index (BMI) [66, 74], smoking [66, 75] and physical inactivity [76].

## Telomere length and telomerase activity – key mediators of mortality and morbidity

Based on the principles of telomere physiology explained earlier, it has been speculated that longer telomeres and high TA are beneficial for healthy aging [77]. In epidemiologic studies, adult men and women with shorter telomeres are characterized by higher mortality rate, which is nearly twice as high as in those with longer telomeres [78, 79]. It has been demonstrated that those with the shortest telomeres were characterized by a higher hazard ratio for all-cause mortality compared to those with the longest telomeres (1.66, 95%CI 1.09–2.53, p=0.018) [80]. In addition, a reduced LTL seems to indicate an existing or an elevated risk for future age-related disease such as CVD, type 2 diabetes mellitus (T2DM), neurodegenerative diseases, osteoporosis and premature aging syndromes [56, 57, 81–83]. Recent clinical association studies unveiled a correlation between leucocytes telomere attrition and clonal hematopoiesis of indeterminate potential (CHIP) [84]. During aging hematopoietic stem cells (hSC) start to accumulate somatic mutations. It can happen that through the accumulation of DNA damage one cell gains a competitive expansion advantage that gives rise to expanded clones of leucocytes with the same mutations. The prevalence of CHIP is very low in those aged <40 years, but can be found in >10% of those aged 70 years and in approximately 20% of octogenarians [84–87]. Individuals who harbour these mutated clones are at higher risk of hematological malignancies, but also several adverse cardiovascular outcomes [88]. In a whole-genome sequencing study, the strongest association of CHIP was found to be an 8 bp deletion in intron 3 of the TERT gene. Accordingly, TLs were observed to be significantly shortened in CHIP carriers [89]. Experimental studies demonstrate a delay in aging and an extended median life span in mice who have been genetically modified with constitutively expressed TERT compared to the respective controls [42, 43]. Moreover, telomerase reactivation reverses tissue degeneration in telomerase deficient mice [90]. On the contrary, constant expression of telomerase has been associated with carcinogenesis and is shown to have detrimental effects [91, 92]. Indeed, 85 to 90% of all human cancers have detectable TA [91]. A pivotal role of telomerase in cancer biology is further highlighted by the fact that inhibition of TA, in telomerase-positive human cancer cells, induces cell death and reduces tumour growth [93–96]. While constant unregulated TA, activation of oncogenes and/or silencing of tumor suppressor genes appears to drive tumour incidence and growth [97], a physiologically regulated telomerase activation appears to have beneficial health effects in mice and humans [59, 98, 99]. Therefore, substantial effort has been invested in the search for lifestyle factors that can modulate TA including nutrition or psychological stress. Based on existing data, also physical activity appears to be an effective way to induce telomerase and to preserve TL [59, 98, 100]. In the following sections, we review existing data on the effects of exercise on aging and in particular on telomere physiology.

## Exercise, health and telomeres

Regular exercise is a well-established approach to reduce the risk of morbidity and premature mortality [13, 101]. Prospective cohort studies demonstrate that men and women who regularly exercise, have a 30% lower all-cause mortality risk than sedentary individuals [13, 101]. In the older persons the beneficial effects of regular physical activity (above 200 minutes a day) are even more pronounced reaching up to >40% mortality risk reduction [101–103]. Some studies have calculated that the gain of life years ranges between 2 to 4 years depending on the individual level of activity [104–108]. Despite strong evidence that supports beneficial health effects through regular exercise, comparability between individual studies is limited because of differences in the composition of study cohorts, exercise protocols and the duration of follow-up [104–108]. However, the pooled analysis of six major cohort studies including 632,091 participants with diverse ethnicity and an average age of 61 years showed that the effect of regular exercise on mortality is dose-dependent and already mild physical activity is associated with a significant reduction of mortality risk and a 1.8-year gain in life expectancy [13]. Metabolic equivalents (MET) are used to compare energy consumption between different activities dividing the actual energy expenditure of a given activity by the energy expenditure at rest [109]. Of note, even intermittent exercise sessions with a limited duration offer considerable health benefits, also in obese individuals and those with major risk factors [110]. The health effects of exercise are not only determined by the frequency and duration of training sessions, but also by the intensity. Vigorous exercise is more effective than mild or moderate exercise in improving cardiorespiratory fitness [111–113]. When adjusted to their specific needs and abilities, even in older individuals, regular physical activity attenuates the age-dependent decline in cardiorespiratory fitness [114], improves mobility and physical functioning [115], and reduces the risk of falls [116].

Besides a substantial reduction of mortality, regular exercise also reduces the incidence and progression of coronary heart disease, hypertension, stroke, diabetes, metabolic syndrome, colon cancer, breast cancer, and depression [101]. When compared to inactive individuals, physically active adults exhibit better cardiorespiratory fitness and muscular strength, a healthier body mass and composition, and a favorable metabolic profile [101]. Furthermore, they report better quality of sleep and health-related quality of life [101]. In a 1-year randomized controlled study regular aerobic exercise (moderate-intensity aerobic exercise 3 days/week at 50–60% of the maximum heart rate reserve for week 1 to 7 and at 60–75% for the remainder of the program of 1 year) was shown to attenuate age-related brain atrophy and improve cognitive function in older individuals [117]. The authors reported that in 120 older persons aged 55–80 years, regular exercise improved memory function and age-related brain atrophy was reversed by approximately 1-2 years [117]. A key mechanism that mediates the neuronal effects of aerobic exercise is the secretion of neurotrophins, and in particular brain-derived neurotrophic factor (BDNF) [118–120].

Despite the existence of robust evidence for multiple health benefits of regular exercise, the underlying mechanisms are insufficiently understood. General key mechanisms that drive the process of aging include the accumulation of genetic damage, epigenetic modifications and shortening of telomeres [121]. It has been speculated that exercise can help preserve TL through the induction of telomerase [99, 122]. In the following section the scientific evidence addressing this concept is reviewed.

### Exercise and telomere biology: animal studies

Although human studies suggest that regular exercise preserves telomeres, they are unable to unveil the underlying mechanisms. Animal models can help to close this gap as they allow to investigate on the mechanistic pathways. At present, only very few animal studies have been performed [59, 98, 123, 124]. It appears that telomeres of murine blood leucocytes and other cell types (e.g. myocardium, liver, aorta) also become shorter with advancing age [59, 98, 123]. However, this process is rather slow and may take between 12 to 18 months. For example, TL of blood leucocytes and cardiomyocytes was comparable in 3-week-old and 6-month-old C57/Bl6 mice, but was significantly reduced after 18 months [59, 98]. Interestingly, the myocardium of these exercising mice also showed increased telomerase and shelterin expression and a reduction of apoptosis and cell-cycle arrest [59, 98, 100]. Regular running exercise has been shown to attenuate the age-related erosion of TL in hepatocytes and cardiomyocytes of CAST/Ei J mice, a wild-derived inbred strain of mice, over a period of 1 year [123]. Moreover, in skeletal muscles and cardiomyocytes the age-related shortening of telomeres is accompanied by a decreased gene expression of the shelterin components TRF1 and TRF2 [123]. Chronic exercise can counteract the reduced expression of shelterins and thus aid to stabilize telomeres [123]. TRF1 and TRF2 protein content showed similar trends that failed to reach significance. In line with these results, Werner et al. reported a persistent up-regulation of cardiac telomere-stabilizing proteins TRF2 and TERT after 6 months of daily running exercise [98]. In parallel, the senescence-related proteins Chk2, p53, and p16 were down-regulated. Together, these effects lead to a substantial reduction of apoptotic cardiomyocytes in the heart of exercising mice. Regular running exercise also ameliorated the cardiotoxic effects of doxorubicin [98]. Overall, experimental studies suggest that the beneficial cardiac effects of regular exercise are primarily mediated by TERT, eNOS, and IGF-1.

Exercise-mediated telomere preservation and other beneficial health outcomes are most likely the result of a cumulative effect over an extended period of time. However, even a single bout of exercise has been shown to increase the protein levels of TRF1 and TRF2 as well as *Pot1a*, but not *Pot1b* gene expression [124]. These changes are accompanied by a greater expression of DNA-repair and -response genes (*Chk2* and *Ku80*) and greater protein content of phosphorylated p38 MAPK [124]. It has been speculated that the rapid increase in shelterin gene expression represents a direct adaptive reaction to the exercise stimulus, which depends on the duration, intensity and type of exercise [124]. In contrast, the fast increase in protein content is probably the result of improved proteostasis rather than increased mRNA translation. The rapid increase of shelterin expression in response to a single exercise session does not necessarily lead to a prompt increase in TA [124]. However, after three weeks of regular training, a persistent upregulation of myocardial TERT expression has been shown by Werner et al. [59, 98]. This activation of TERT appears to be essential for the cardioprotective effects of physical activity.

Although existing evidence is rather limited, available data suggest that exercise induces an immediate short-lived regulatory response in shelterin mRNA expression, but only a continuous stimulation over an extended period of time results in a preservation of telomeres and delays cellular aging. Furthermore, regular exercise is directly involved in the establishment of an anti-apoptotic and anti-senescent cellular environment through up-regulation of genes implicated in the DNA damage response and repair, including *Ku70/Ku80* and down-regulation of *p16*, *p53* and *Chk2* [98, 123].

Interestingly, the myocardium of exercising mice showed increased telomerase and shelterin expression and a reduction in apoptosis and cell-cycle arrest [59, 98, 100].

### Exercise and telomere biology: human studies

The first study to explore the relationship between exercise and TL in humans was conducted by Cherkas et al. In a cross-sectional survey of 2401 white men and women they showed that LTL was positively associated with higher physical activity levels [125]. Similar results were reported by Du et al. analyzing 7,813 adult women from the Nurses’ Health Study, where even moderate amounts of activity were associated with longer telomeres [126]. In 5823 adult men and women of the National Health and Nutrition Examination Survey (NHANES 1999-2002) Tucker et al. showed that average LTL decreases by 15.6 bp per year of chronological age [127]. Individuals with higher levels of physical activity had substantially longer telomeres in peripheral blood leucocytes, corresponding to a gain of biological age of approximately 9 years [127]. All these epidemiologic studies are limited by their cross-sectional nature and the fact that physical activity is self-reported. However, several smaller studies support the concept of telomere preservation by regular exercise [128–130]. In a comparison of telomere biology in young and middle-aged endurance athletes with sedentary controls, Werner et al. demonstrated that regular endurance training is associated with a reduction in leucocyte telomere erosion [59]. In their study, LTL of middle-aged athletes was preserved at the level of young controls. In contrast, LTL of middle-aged controls was approximately 30-40% lower than in young controls and thus, demonstrating an age-related attenuation. The preservation of TL was confirmed by two independent methods, qPCR and flow-FISH. Furthermore, when compared with untrained individuals, athletes showed increased TA and expression of telomere-stabilizing shelterin proteins, such as TRF2. The effects on telomere biology were accompanied by a pronounced inhibition of the DNA damage checkpoint kinase (*Chk2*) and the regulators of cell-cycle progression and survival, termed *p16* and *p53* [59]. In line with these results, Denham et al. analysed LTL and the expression of telomere-regulating genes in 61 Australian endurance athletes and 61 healthy controls [51]. LTL in athletes was 7.1% (208-416 nucleotides (nt)) higher than in sedentary controls. In addition, athletes showed a higher expression of *TERT* and *TPP1* mRNA expression. Interestingly, resting heart rate emerged as an independent predictor of LTL, *TERT* and *TPP1* mRNA expression in this study. Denham et al. also showed that training volume determines the effect of exercise on telomere biology with the greatest effects seen in the most active athletes. A much smaller study from Østhus et al. showed greater LTL in older endurance athletes than in individuals of the same age with a medium level of activity [131]. However, young individuals with high and low activity levels showed no difference in LTL. On the molecular level telomere-associated genes, including *TERT*, *TERF2IP* (which encodes RAP1), Sirtuin-6 (*SIRT6*) and TATA-box binding protein (*TBP*) and miRNAs that target these genes are upregulated after a single running session of 30 minutes at 80% of peak oxygen uptake (VO2Peak). The analysis of white blood cells from 22 healthy male volunteers, immediately after and 60 min after exercise, showed that 56 miRNAs were differentially regulated post-exercise (FDR <0.05) and that 4 of these (miR-186, miR-181, miR-15a and miR-96) potentially target telomere-associated mRNA species [132].

Although cross-sectional observation studies suggest that regular exercise preserves TL through an activation of telomerase, experimental and prospective studies are necessary to proof causality. A recent study in 124 healthy previously inactive individuals explored the effects of regular endurance training, intensive interval training and resistance training over a period of 6 months [99]. Participants trained 3 times per week for 45 min. Compared to non-exercising controls, TA in blood mononuclear cells was up-regulated 2 to 3-fold in the endurance- and interval-training groups, but not in the resistance-training group. The activation of telomerase was accompanied by longer telomeres in lymphocytes, granulocytes, and leucocytes. In addition to this training study, Werner et al. also explored the effects of a single bout of exhaustive exercise using a stepwise ramp protocol on a treadmill. When compared to baseline, CD14+ and CD34+ leucocytes collected after exercise, exhibited increased TA, which was still measurable 24h-post exercise. IGF-1, a potential mediator of the exercise-induced activation of telomerase [59], showed a biphasic response. However, after the 6-month training program, IGF-1 was comparable to baseline levels. Furthermore, blood collection was performed from 48 hours to 7 days after the last exercise session. This suggests that whilst the exercise-induced effects on telomere biology are of short duration, any health benefit is the result of a cumulative effect achieved by regular training. The beneficial effects of long-term exercise on TL and TA have also been shown by Melk A et al. in 59 healthy middle-aged men with former sedentary lifestyles [133]. Besides the secretion of IGF-1, another putative hypothesis to explain the exercise-induced activation of telomerase with subsequent telomere elongation is the release of nitric oxide (NO) as a result of increased vascular shear stress [99]. Endothelial NO synthase and TA appear to be linked in a signalling pathway that mediates vascular protection [59].

Despite robust evidence from cross-sectional and prospective intervention studies, not all previously published analyses support a relationship between exercise and telomere biology [134–139]. For example, a comparison of 17 marathon runners and 19 healthy sedentary controls reported no difference in LTL [136]. Similar findings were reported by Song et al. in 84 healthy volunteers [135]. Finally, in a cross-sectional and longitudinal analyses of 582 older adults, Soares-Miranda et al. found no consistent relationship between physical activity and LTL [137]. Only some general functional measures, such as walking distance and “chair test performance”, were cross-sectionally related to LTL. In addition, changes in leisure-time activity and in “chair test performance” altered the change in LTL over time. Results from the “Berlin Aging Study” suggest that, in adult men aged over 61 years, long periods of physical activity are necessary for the prevention of telomere shortening (at least 10 years), with intensive sports activities having the greatest effect [140]. This concept is confirmed by a study from Lane et al. where former elite athletes were found to have comparable LTL to age-matched, sedentary individuals [141].

Some researchers suggest that the relationship between LTL and exercise is U-shaped [51, 142, 143]. For example, Savela et al. analysed physical activity levels, LTL and the proportion of short telomeres in 204 randomly selected survivors of the “Helsinki Businessman Study”. Moderate physical activity was associated with the longest mean LTL. A cross-sectional comparison of endurance athletes and healthy controls provides additional support that moderate amounts of exercise training protects against biological aging, while higher amounts may not elicit additional benefits [51].

In summary, the evidence implies that the protective effects of exercise require a rather long-time span and continuity in order to become evident.

### Differential effects of exercise modalities on telomere biology

As reported above, it is not clear whether exercise can preserve or increase TL. The controversial results may be explained, in part, by the fact that “exercise” is a general term that includes many different types of physical activities, such as running, swimming, dancing, weightlifting, ball sports and others. Therefore, the question arises whether different exercise modalities exert differential effects on telomeres? Most existing studies have investigated the effects of endurance exercise [59, 128, 144], in particular running and cycling, or mixed exercise regimens [126, 129]. However, in most epidemiologic studies, physical activity was self-reported [125, 145, 146]. To date, only one study directly compared the effects of different exercise modalities on telomere biology [99]. This randomized controlled trial showed that only endurance and high-intensive interval training, but not resistance training, increased TA and LTL in middle-aged healthy individuals. All intervention groups performed 3 exercise sessions per week with a duration of 45 min for 6 months. In an analysis of the NHANES (1999-2001), different types of self-reported leisure time activities were assessed, and only moderate/vigorous physical activity was significantly associated with LTL [147]. A lack of resistance training to preserve TL has also been observed in a small cross-sectional study that compared power lifters with healthy, active individuals with no history of strength training [148]. In summary, there is insufficient data to judge if different training modalities exert differential effects on telomeres, telomerase and shelterin expression. However, existing studies suggest that aerobic endurance exercise, but not resistance training, is helpful to preserve TL, at least in leucocytes.

## Mechanistic considerations

**Figure 1:**
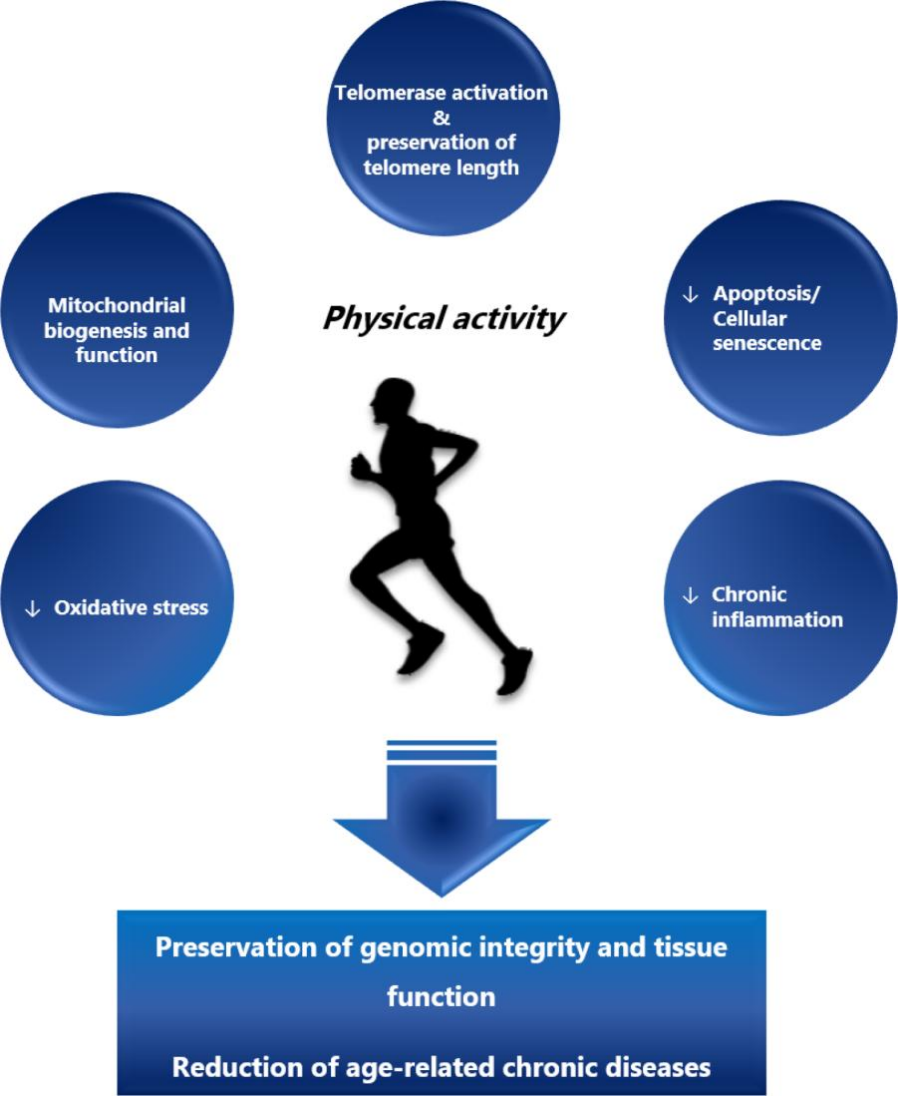
**The beneficial effects of regular physical activity.** Regular physical activity exerts its beneficial effects through activation of telomerase, preservation of telomere length and improved mitochondrial biogenesis and function. On the cellular level these effects lead to the reduction of apoptosis, cellular senescence and oxidative stress, lowering the subsequent multi-system chronic inflammation. In summary, regular physical activity is a means to preserve genomic integrity and tissue function and reduce the onset of age-related chronic diseases.

Besides the preservation of telomeres, several other mechanisms have been proposed to contribute to the anti-aging effects of physical activity (Figure 1). Regular endurance exercise over 5 months improved mitochondrial biogenesis and morphology in skeletal muscles and other organs including lungs and heart in mtDNA mutator mice (animals with accelerated rates of mitochondrial DNA mutation). As a result, exercise delayed the age-related degeneration process of multiple organs, increased mobility, and attenuated telomere shortening [149, 150]. As noted, exercise contributes to an increased shelterin expression via upregulation of p38 MAPK and a subsequent regulation of several transcription factors [123], including the upstream transcription factors of the PGC-1α gene. PGC-1α is a pleiotropic protein involved in cellular energy metabolism [151, 152] that has also been linked to aging [153]. During endurance exercise and caloric restriction, PGC-1α is activated by adenosine monophosphate-activated protein kinase (AMPK), accumulates in the nucleus through sirtuin 1-dependent deacetylation and acts as a co-activator for other transcription factors [153, 154] including nuclear respiratory factor 1 (NRF-1), a regulator of mitochondrial biogenesis [155, 156]. Age-dependent telomere shortening contributes to mitochondrial and genomic DNA damage via activation of p53 and down-regulation of PGC-1α/β [157]. Recently, de Carvalho Cunha et al. have demonstrated that exercise regulates *p53* and *Chk2* in an intensity-dependent fashion, with high intensity endurance exercise being more effective in downregulating *p53* than low intensity exercise [158]. Moreover, high intensity training appears to be more effective in enhancing antioxidant defense, AMPK and PGC-1α expression [159].

Today, only very few animal studies have explored the mechanisms behind the exercise-mediated preservation of telomeres [59, 98, 123, 124]. Although these studies seem to confirm the results obtained in human studies, many mechanistic aspects remain to be clarified. Therefore, future research is needed to improve our understanding on the effects of exercise on telomere biology and genomic aging.

## Analytical aspects

Despite robust evidence linking leucocyte telomere shortening with aging and age-related diseases, the measurement of LTL is not yet used clinically. Several unresolved pre-analytical, analytical and post-analytical aspects have hampered the transition of this promising marker from research laboratories into routine diagnostics. From a pre-analytical point of view the pronounced inter-individual variability of LTL [78, 160] and leucocyte telomere shortening [161] complicate a meaningful interpretation of individual results. Aviv et al. have shown that amongst young adults LTL changes between -240 and +12 bp per year. As previously discussed, telomere shortening throughout life is not a linear process instead it is most pronounced during the period of rapid somatic growth in the first two years of life [34, 35, 38, 39]. In addition, young individuals with either longer or shorter than the average TL tend to maintain that classification throughout the rest of their life [162, 163]. Together, these unresolved pre-analytical issues have prevented a consensus amongst researchers and clinicians as to when measurement of TL is meaningful and might provide a benefit for the individual.

Another major concern is the appropriate sample matrix. Variable proliferation rates lead to vastly different TLs amongst several tissue types [35]. In different organs, from the same individual, TL can differ by factor 6 and more [35]. It also appears that TL within the same organ varies substantially, and consequently results depend on the site of sample collection. Only few studies have investigated the distribution of TL in different organs from the same donor. Perhaps normalizing LTL for TL of a post-mitotic tissue like fat or skeletal muscles might provide a better understanding of leucocyte telomere dynamics during aging [164]. Dlouha et al. measured telomere length in twelve human tissues (peripheral blood leukocytes, liver, kidney, heart, intercostal skeletal muscle, subcutaneous and abdominal fat) from dead human donors with a wide age range (29 weeks to 88 years). They found an inverse relationship between relative telomere length (rTL) and donor age, with the longest rTL detected in the youngest [35]. TL was significantly higher in blood compared to the majority of tissues but not different compared to adipose and renal tissue. The largest interindividual variability was observed in leucocytes and kidney [35]. Nonetheless, these results were confounded by the small number of donors and their variable health status. Up until now, little is known about the effect of injury and physical activity on telomere dynamics in human skeletal muscle. A recent study assessed whether aerobic capacity was associated with TL in skeletal muscles and leucocytes and whether TL is associated in these two tissues, across a wide age range (18–87 years). The findings support a correlation between LTL and mean skeletal muscles telomere length indicating that individuals with short (or long) telomeres in one tissue also display short (or long) telomeres in another tissue. However, skeletal muscle TL was not associated with age, and aerobic capacity was not associated with longer telomeres in either leukocytes or skeletal muscles [139]. Therefore, more studies are needed to consolidate our knowledge about tissue specific differences in telomere dynamics.

To avoid invasive sample collection and regional variability of TL in solid organ tissues, blood leucocytes have been proposed as an alternative matrix for telomere analysis. Blood can easily be collected multiple times and LTL, at least theoretically, mirrors telomere dynamics in hematopoietic stem cells (hSC) and is an index of hSC reserve [165, 166]. However, blood leucocytes represent a heterogeneous cell population including monocytes, granulocytes and lymphocytes. The composition of this population is highly variable depending on stressors i.e. exercise, nutrition, smoking, psychological stress and others. These stressors can trigger a redistribution of leucocytes from immune reservoirs to the circulation and peripheral tissues [167]. As a result, the percentage of neutrophil granulocytes can range from 40 to 70% of the entire leucocyte count. Compared to many other cell types, neutrophils have a very short lifespan of 1-3 days. Therefore, it is not surprising that LTL exhibits by far the highest intra- and inter-individual variability amongst all sample types [35]. Conditions, such as CHIP, which arise from leucocyte precursor cells, may also influence the distribution of LTL and thus hamper the interpretation of LTL results. None withstanding the potential association between LTL and CHIP, which is primarily based on observational data, variable telomere attrition rates between individuals and amongst different solid tissues remain a major issue when interpreting the results of TL measurements. Therefore, more experimental data are needed to consolidate our knowledge about the relationship between TL in leucocytes and different solid tissues in the context of CHIP and other TL modifying conditions [168]. In summary, our present knowledge is insufficient to judge the validity of LTL as marker of biological age and as prognostic tool for poor outcomes and shorter DALYs in clinical settings. Furthermore, it is not clear how telomere dynamics of peripheral blood leucocytes reflect pathophysiological changes in individual organs.

Besides the aforementioned pre-analytical issues, there are also analytical aspects that hamper a wider use of TL analysis. Existing methods are quantitative PCR (qPCR), Terminal Restriction Fragment (TRF) analysis by Southern blot, fluorescence in situ hybridization coupled with flow cytometry (flow-FISH), Single Telomere Length assay (STELA), Universal STELA, and Telomere Shortest Length Assay (TeSLA). Although all these methods analyse TL, the information they provide is substantially different and the results are not directly comparable [169]. Briefly, the qPCR assay is most frequently used in epidemiologic studies because it is easy to perform, requires small amounts of DNA and allows high throughput. The method provides a relative TL (T) compared to a single copy gene (S) and results are expressed as a T/S ratio. Information about the distribution of short and long telomeres, as well as differences between individual chromosomes and cells cannot be obtained. TRF is considered the “gold standard” for TL analysis that measures the intensity of telomere smears to determine an average TL. However, applicability of this method is limited by the requirement of large amounts of DNA (approx. 3 μg) and a relatively laborious and time-consuming assay procedure, additionally with this technique very short telomeres (approx. 2 kb or less) are difficult to detect. Although reproducibility within the same laboratory can be rather good, results cannot easily be compared between laboratories. However, commercial TRF kits are now available and may help to improve inter-laboratory comparability. TL of peripheral blood leucocytes can also be measured by fluorescence in-situ hybridization (FISH) based methods. FISH based methods produce very reliable results, but are laborious and require expensive instrumentation [169]. Q-FISH expresses TL as relative fluorescence units. With the help of TRF measured standards absolute TLs can be derived. With this technique it has been shown that the shortest telomeres determine cell viability and chromosome stability [170–172]. Reliable measurement of the shortest telomeres might open new possibilities for the assessment of biological age, the determination of individual risk for age-related degenerative disease and patient management. Finally, TeSLA assay, requires only small amounts (<1μg) of DNA and allows the unbiased measurement of TL distribution [173]. A wider use of TeSLA is hampered by its low throughput. Furthermore, very long telomeres, such as in inbred strains of mice, are not captured by this method. For a more comprehensive overview on the various techniques we refer to a recent review from Lai et al. [169]. Yet, for the measurement of TA the commonly used assay remains the Telomere-Repeat Amplification Protocol (TRAP), a two-step procedure composed of telomerase mediated primer extension and PCR-based detection of extended products. This method has been further adapted to combine TRAP and droplet digital PCR (ddTRAP), thus increasing the sensitivity, repeatability and throughput of the assay. The specifics of the latter are reviewed by Ludlow at et al. [174, 175]. More laborious and not clinically used methods to detect TA include PCR-free assays such as electrochemical assays, optical assays, and signal-transduction assays. However, all of them must be optimized to improve throughput and sensitivity and need special instrumentation to be performed [176].

In summary, telomere length and TA are almost exclusively measured in research laboratories. Sample matrix and analytical procedure should be carefully chosen for the intended use, and analyses should be performed by sufficiently trained staff.

## CONCLUSIONS

Telomere research has gained much attention in the previous decade for its potential use and promise as a future therapeutic target, disease management and measurement of genomic aging. Interventions, such as physical activity that target the deleterious processes of aging have concomitantly created interest in the area of lifestyle and aging related research. Largely, the available physical activity data do not exclude that an association between regular exercise and TL exists. However, to date, the observed results from human studies are skewed largely by associations and observational or cross-sectional data. In light of the limited data, available evidence suggests altogether, that regular, and consistent physical activity over an extended period of time may assist with preservation of telomeres and cellular aging. Nevertheless, conflicting and a lack of consistent findings from the existing evidence, and particularly from the few available mechanistic studies means there is much more to explore and understand, prior to measurements such as TL will be adopted clinically.

Considering the above, future research should be focused on 1) developing more experimental data to further elucidate and confirm the relationship and mechanistic pathways between physical activity, aging and telomere biology, 2) investigating the effects of the use of different exercise modalities and intensities on telomeres and 3) further determining if these effects are tissue-specific or systemic.
